# Gingival fibroblasts resist apoptosis in response to oxidative stress in a model of periodontal diseases

**DOI:** 10.1038/cddiscovery.2015.46

**Published:** 2015-11-09

**Authors:** R Cheng, D Choudhury, C Liu, S Billet, T Hu, NA Bhowmick

**Affiliations:** 1 State Key Laboratory of Oral Diseases, West China Hospital of Stomatology, Sichuan University, Chengdu, China; 2 Cedars-Sinai Medical Center, Los Angeles, CA, USA; 3 Affiliated Hospital of Stomatology, Zhejiang University, Hangzhou, China

## Abstract

Periodontal diseases are classified as inflammation affecting the supporting tissue of teeth, which eventually leads to tooth loss. Mild reversible gingivitis and severe irreversible periodontitis are the most common periodontal diseases. Periodontal pathogens initiate the diseases. The bacterial toxin, lipopolysaccharide (LPS), triggers the inflammatory response and leads to oxidative stress. However, the progress of oxidative stress in periodontal diseases is unknown. The purpose of this study is to examine oxidative stress and cell damage in gingivitis and periodontitis. Our results showed that LPS increases reactive oxygen species (ROS) accumulation in gingival fibroblast (GF). However, oxidative stress resulting from excessive ROS did not influence DNA damage and cell apoptosis within 24 h. The mechanism may be related to the increased expression of DNA repair genes, Ogg1, Neil1 and Rad50. Detection of apoptosis-related proteins also showed anti-apoptotic effects and pro-apoptotic effects were balanced. The earliest damage appeared in DNA when increased *γ*H2AX, an early biomarker for DNA damage, was detected in the LPS group after 48 h. Later, when recurrent inflammation persisted, 8-OHdG, a biomarker for oxidative stress was much higher in periodontitis model compared to the control *in vivo*. Staining of 8-OHdG in human periodontitis specimens confirmed the results. Furthermore, TUNEL staining of apoptotic cells indicated that the periodontitis model induced more cell apoptosis in gingival tissue. This suggested GF could resist early and acute inflammation (gingivitis), which was regarded as reversible, but recurrent and chronic inflammation (periodontitis) led to permanent cell damage and death.

## Introduction

Periodontal disease is an inflammation of the soft and hard tissues that support the teeth. These tissues include the gums, the periodontal ligaments, and the alveolar bones that surround the teeth. Gingivitis and periodontitis are the most common periodontal diseases. The inflammation of the gums surrounding the teeth is defined as gingivitis, which is reversible after timely and effective tooth clean (including tooth brushing and periodontal scaling). However, chronic and recurrent gingivitis ultimately result in receding gums and can cause irreversible damage to alveolar bone, which is defined as periodontitis.^1^ Thus, the mechanism in maintaining the reversible capacity of gingivitis is of great importance for disease control.

Bacteria are the main cause of periodontal diseases. The major component of Gram-negative bacterial outer membrane, lipopolysaccharide (LPS), can trigger the inflammatory response, leading to the release of large numbers of inflammatory mediators, including interleukins, chemokines, adhesion molecules and reactive oxygen species (ROS).^2–5^ These pro-inflammatory mediators are required for immune defense against bacteria, but when the activity is uncontrolled, ROS accumulation is observed. ROS, including superoxide radicals, hydrogen peroxide and hydroxyl radicals, are involved in a variety of chronic diseases, such as atherosclerosis, pulmonary toxicity, macular degeneration, cataractogenesis, diabetes, cancers and renal fibrosis.^6^ Bacteria-induced oxidative stress can cause tissue damage, vascular barrier dysfunction and perpetuation of the inflammatory cascade.^7^

Oxidative stress is recognized to be involved in periodontitis.^8,9^ To test the hypothesis that LPS-induced oxidative stress can have differential consequences when involved in gingivitis and periodontitis, we simulated acute gingivitis and chronic periodontitis to study the oxidative stress and cell death that ensued.

## Results

### LPS induces oxidative stress and DNA damage in gingival fibroblast cells

**Figure 1:**
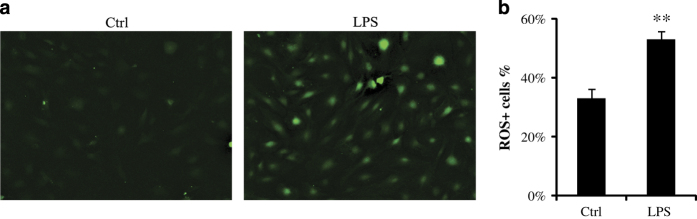
Gingival fibroblast was treated with LPS (1 *μ*g/ml) for 24 h. Reactive oxygen species (ROS) were determined by DCFH-DA staining. (**a**) LPS increases ROS accumulation in gingival fibroblast (magnification ×100). (**b**) The ROS-positive cells were counted by flow cytometry. The experiment was repeated three times with similar results (Ctrl, control; **P<0.01).

**Figure 2:**
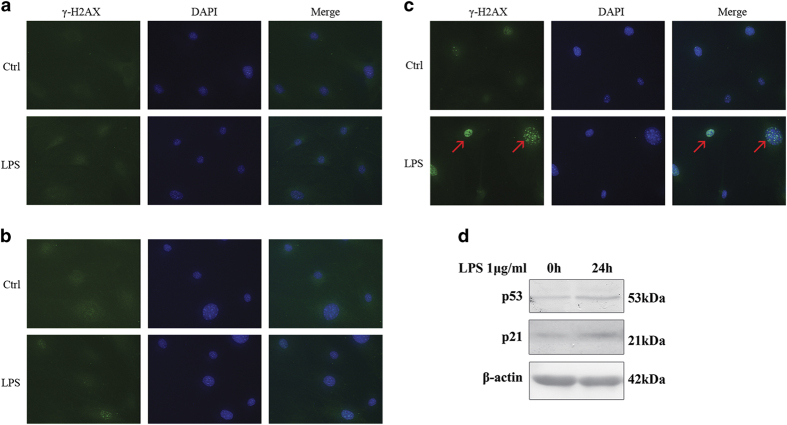
DNA damage appeared after stimulation of LPS for 48 h. Immunofluorescent localization of *γ*H2AX in gingival fibroblasts was performed following 1 *μ*g/ml LPS treatment for 6, 24 and 48 h. (**a** and **b**) *γ*-H2AX was not detected in 6 and 24 h, respectively. (**c**) But *γ*-H2AX was increased in cell nuclei by LPS in 48 h (red arrow; Ctrl, control; magnification ×400). (**d**) LPS (1 *μ*g/ml) increased p53 and p21 in 24 h.

Acute gingivitis mouse model has not been acknowledged. In this study, gingival fibroblasts were stimulated by LPS *in vitro* for 24 and 48 h, to simulate acute and early inflammation. It is evident that LPS has the ability to induce ROS accumulation and promote pro-inflammatory cytokine expression.^4^ ROS buildup in gingival fibroblast after 24 h of LPS stimulation was supported by observed DCFH-DA staining (Figure 1). ROS can directly result in DNA damage. DNA damage induces the phosphorylation of histone variant H2AX (*γ*H2AX) to recruit proteins to repair double-stranded DNA breaks.^10,11^ Figure 2 illustrates a time-dependent accumulation of DNA double-stranded breaks, detectable after 24 h of LPS-induced oxidative stress. The effects of ROS on gingival fibroblast DNA was evident by 48 h. DNA damage can potentiate apoptosis and senescence as a factor of p53 status.^12^

Figure 2d showed p53 had increased as a response to DNA damage in 24 h. In addition, the levels of p21, a p53 transcriptional target acting as cyclin-dependent kinase inhibitor, were also found to increase.^13^ Since these primary gingival fibroblastic cells have intact p53, the expectation was the apoptotic death of DNA-damaged cells. However, the differences in short *versus* long exposure to LPS have not been studied in gingival fibroblasts.

**Figure 3:**
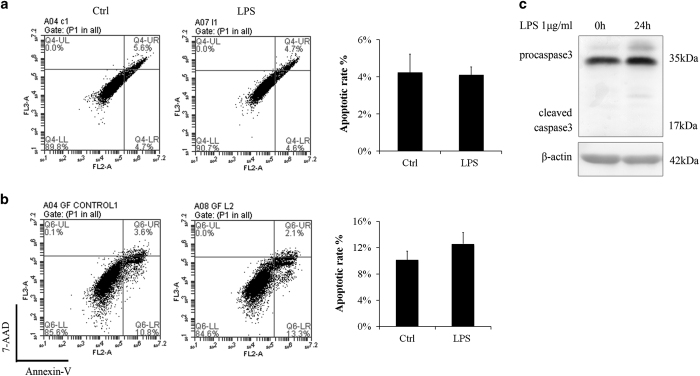
Even though ROS was increased and DNA damage appeared, cell apoptosis rate were not changed by LPS. Annexin V/7-AAD staining followed by flow cytometry was conducted to study the apoptotic rates. (**a**) LPS (1 *μ*g/ml) had little influence on apoptosis rate and cell cycle in 24 h. The experiment was repeated three times and statistical analysis was performed. (**b**) Even in 48 h, LPS (1 *μ*g/ml) had little influence on apoptosis rate (Ctrl, control). The experiment was repeated three times. (**c**) Cleaved caspase-3 is not obvious after stimulation of LPS (1 *μ*g/ml) for 24 h.

Cell apoptosis is a possible consequence when oxidative stress and DNA damage persist. To study the short-term ROS effect, we exposed gingival fibroblasts to LPS for as long as 48 h *in vitro*. Cell apoptosis was examined by flow cytometry. The results showed that apoptosis rates have no difference in the 24- and 48-h LPS groups compared to control treatments (Figures 3a and b). Detection of caspase-3 confirmed cell apoptosis is not detectable in 24-h LPS group (Figure 3c).

### DNA damage and apoptosis in chronic periodontitis

In a long-term study, an experimental periodontitis *in vivo* model was used where gingival tissue of mice was administered LPS for 3 weeks.^14^

**Figure 4:**
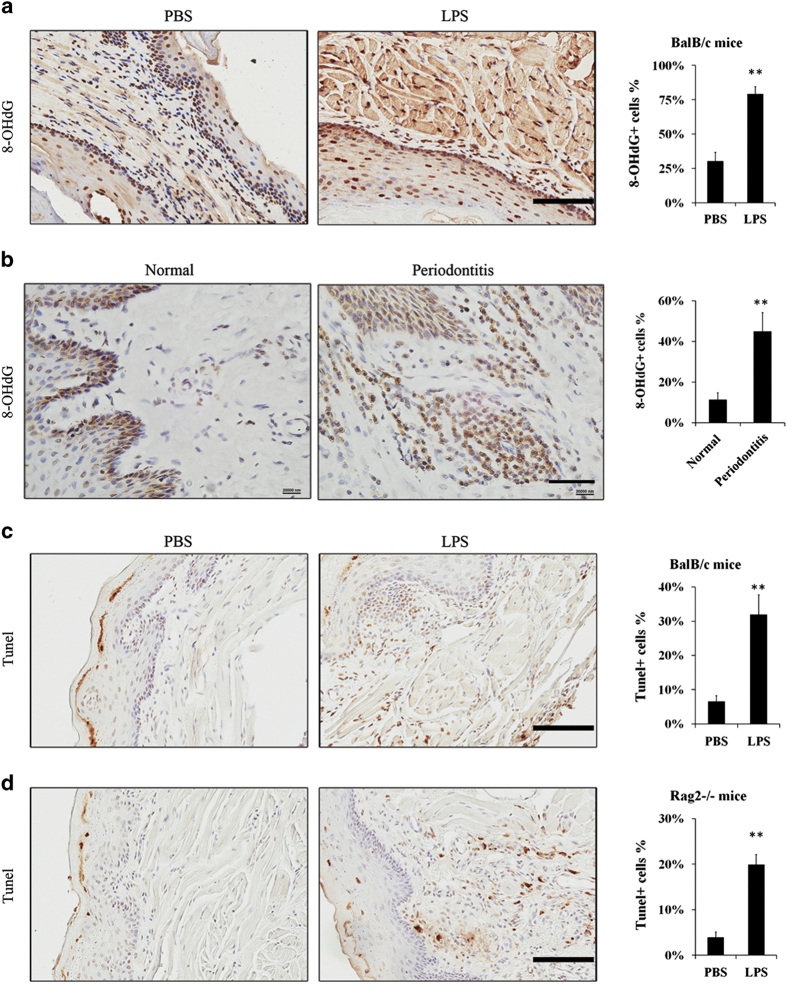
Chronic periodontitis-induced apoptosis and DNA damage in gingiva. Approximately, 10 *μ*g LPS in 10 *μ*l PBS or 10 *μ*l PBS vehicle were injected into mandibular buccal gingiva two times per week for 3 weeks to establish experimental periodontitis models or PBS vehicle models. (**a**) There were higher expressions of 8-OHdG in the nuclei in BalB/c mice periodontitis models compared to the PBS vehicle models (scale bar, 100 *μ*m). (**b**) Paraffin-embedded human gingival samples were stained for 8-OHdG by IHC (other two healthy and four periodontitis specimens were shown in Supplementary Figure 1; scale bar, 50 *μ*m). The percentage of positive cells was counted from four microscopic fields per sample. (**c**) TUNEL staining showed more apoptotic cells in BalB/c mice periodontitis model, especially in submucosal tissue (scale bar, 100 *μ*m). (**d**) TUNEL staining in *Rag2^−/−^* mice periodontitis model (scale bar, 100 *μ*m). The percentage of positive cells (**a**, **c** and **d**) was counted from three to four mice samples and four microscopic fields per sample (**P<0.01).

8-OHdG is one of the predominant forms of free radical-induced DNA oxidation, and has therefore been widely used as a biomarker for oxidative stress.^15,16^ The expressions of 8-OHdG were mainly in the nuclei compared to the cytoplasm. Of note, cytoplasmic 8-OHdG can be attributed to mitochondrial DNA damage and mitophagy, which was not evident in our studies. Figure 4a illustrates epithelia, fibroblasts and muscle cells had high 8-OHdG expressions. More cells in submucosal tissue had high expression of 8-OHdG in the chronic LPS treatment group than the control, PBS-treated group (Figure 4a). The results were confirmed at periodontitis diseased sites in human gingival tissues. Figure 4b showed five periodontitis samples had more submucosal cells with positive staining of 8-OHdG than three healthy gingival tissue. It suggested that DNA oxidation was aggravated by LPS-induced periodontitis.

To study the long-term effect to oxidative stress, cell apoptosis in experimental periodontitis was examined by TUNEL staining. Contrary to short-term results, the number of TUNEL-positive cells was higher in experimental periodontitis model than the control (Figure 4c). It suggested apoptosis of gingival fibroblast cells occurred in a long and chronic process. The profound immune response to LPS treatment was next limited by the use of *Rag2^−/−^* mice in the identical periodontitis model. *Rag*-2-deficient mice fail to produce mature B and T lymphocytes.^17^ The *Rag2^−/−^* periodontitis mouse model had greater number of apoptotic cells compared to the PBS-treated control (Figure 4d). It suggested that LPS could directly induce apoptosis in gingival fibroblast cells in a cell intrinsic manner.

Contrary to short-term stimulation of LPS, the results showed LPS-induced experimental periodontitis could lead to DNA damage and apoptosis. The results suggested gingival fibroblast could survive when exposed to LPS in the short-term period. It indicated periodontal disease (gingivitis) may not cause severe damage.

### The anti-apoptotic mechanism of gingival fibroblast in gingivitis

**Figure 5:**
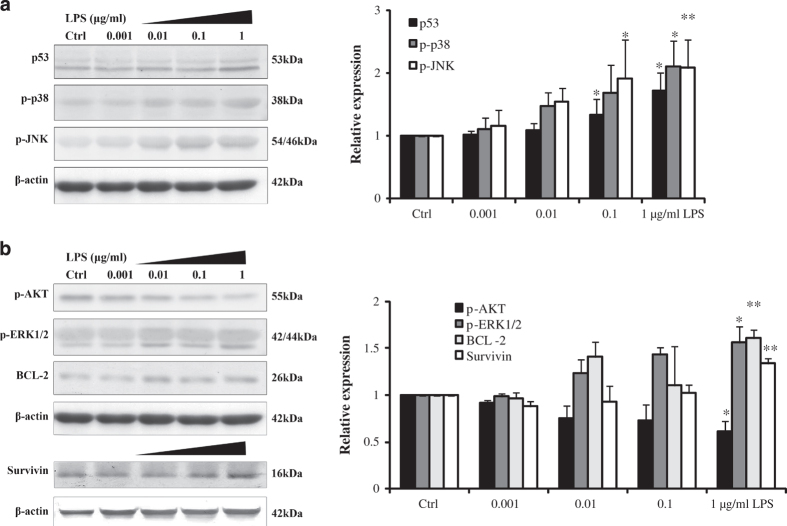
The mechanisms of gingival fibroblast in cell apoptosis resistance. (**a**) Effects of LPS on the expressions of pro-apoptotic proteins and (**b**) anti-apoptotic proteins *in vitro*. Gingival fibroblasts were treated with different doses of LPS for 24 h (0, 0.001, 0.01, 0.1 and 1 *μ*g/ml LPS). The results showed that LPS increased phosphor-ERK1/2 (p-ERK), phosphor-p38 (p-p38), phosphor-JNK (p-JNK), p53, BCL-2 and survivin in a dose-dependent manner. Simultaneously, phosphor-AKT (p-AKT) decreases in a dose-dependent manner. Error bars represent the S.D. of three different experiments (*P<0.05; **P<0.01).

**Figure 6:**
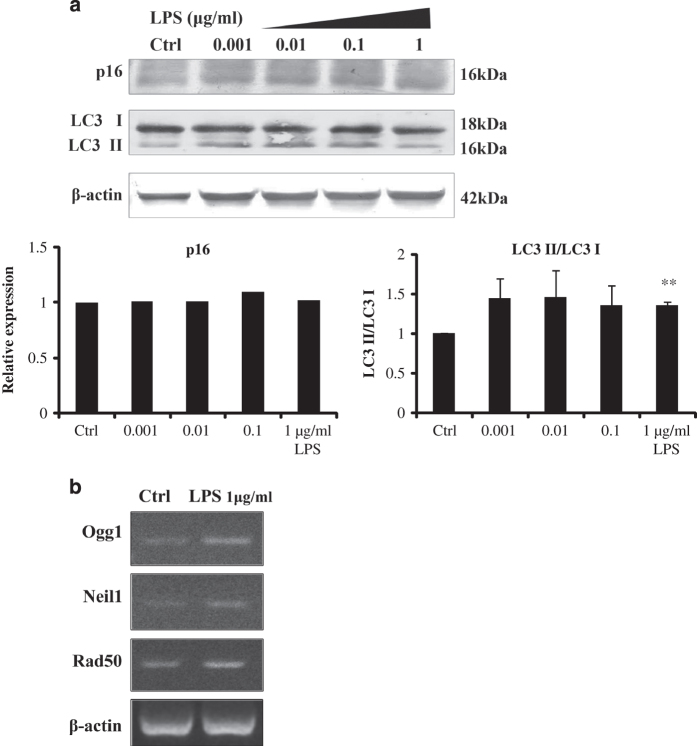
Other possible mechanisms in cell survival process. (**a**) Gingival fibroblasts were treated with different doses of LPS for 24 h (0, 0.001, 0.01, 0.1 and 1 *μ*g/ml LPS). LPS increased the ratio of LC3 ІI/LC3 I, indicating autophagy was involved. However, the protein level of p16 had not changed. Error bars represent the S.D. of three different experiments (**P<0.01). (**b**) Stimulation of LPS for 24 h increased the mRNA expressions of DNA repair genes (Ctrl, control).

To elucidate the mechanism of how gingival fibroblast could resist toxic effects of LPS, we examined apoptotic effectors. Not surprisingly, the stress kinases, p-JNK and p-P38, were activated in a dose-dependent manner of LPS (Figure 5). The concomitant DNA damage associated increase in the expression of p53 was also not surprising. But, the generally considered pro-survival kinase, p-AKT, was decreased in increasing LPS concentrations. However, in contrast to its ability to inhibit apoptosis induced by multiple apoptotic stimuli, decreased AKT could inhibit ROS-mediated apoptosis.^18^ The elevation of anti-apoptotic, BCL-1 and ERK activation likely countered apoptotic effects. The upregulation of anti-apoptotic protein expression of survivin further revealed the gingival fibroblasts means of survival (Figure 5b). Measures of DNA damage repair genes were taken, Ogg1 and Neil1 belong to DNA glycosylase enzymes that repair DNA damages caused by oxidative stress, Rad50 is involved in DNA double-strand break repair, were all found to be upregulated under short-term stimulation of LPS (Figure 6b).

Since apoptosis was not detected, senescence and autophagy might be the consequences of DNA damage. Figure 2d had suggested p21-mediated senescence was involved. Another maker of senescence, p16 and marker of autophagy, LC3 was examined. The results suggested autophagy, but not p16-mediated senescence was involved in cell survival and potential regenerative process (Figure 6a).

## Discussion

Oxidative stress is a condition in which pro-oxidant–antioxidant balance in the cell is disturbed. Under severe oxidative damage, cell death is the ultimate result. A number of studies have shown that oxidative stress could cause cell apoptosis. This study showed that LPS was able to increase ROS accumulation in gingival fibroblast cells in 24 h. It suggested oxidative stress occurred in early inflammation. Bacterial endotoxin LPS induces the production ROS under *in vivo* and *in vitro* conditions. Multiple studies have demonstrated that LPS significantly increases intracellular ROS production in various cell types, including vascular smooth muscle cells, macrophage and vascular endothelial cells.^19–21^ A study even revealed that LPS induced significant ROS levels in human dental pulp cells.^22^ LPS from *Porphyromonas gingivalis* upregulated ROS in periodontal ligament fibroblasts.^23^ As a result of ROS accumulation, the toxicity of LPS might induce oxidative stress on gingival fibroblast cells.

For the measurement of the damage of oxidative stress, products of radical damage to DNA, lipids, proteins and protective species are good markers of ROS-induced oxidative stress.^24^ In this study, *γ*H2AX was detected for early DNA damage; 8-OHdG was detected for oxidative damage to DNA. The results showed mild DNA damage was observed only after 48 h. It seems that DNA damage could be resisted in 24 h (the early stage of acute gingival inflammation). However, high levels of 8-OHdG was found in mouse periodontitis models and human periodontitis samples (Figure 4). The results were coincident with previous studies that have suggested periodontitis-induced oxidative stress. In experimental periodontitis, the expressions of 8-OHdG level and nitrotyrosine in fibroblasts or leukocytes have been demonstrated.^8,9^ Experimental periodontitis also increased plasma 8-OHdG levels and the level of serum reactive oxygen metabolites in rat periodontitis models.^25,26^ There was no doubt that oxidative stress existed in periodontitis, but our results suggested that DNA damage was continuously accumulated when inflammation persisted.

When oxidative stress persists, cell apoptosis increases as well. A large number of studies have shown that oxidative stress could cause cellular apoptosis.^27–29^ Previous studies have observed apoptosis in chronic periodontitis as well. In human periodontitis tissue, most of the TUNEL-positive cells belonged to neutrophil cell populations.^30,31^ Periodontitis and diabetic periodontitis also increased apoptosis of fibroblasts, osteoblasts and osteocytes.^32^ Our study confirmed that experimental periodontitis-induced apoptosis of fibroblasts by using immune complete and deficient mice.

Compared with these periodontitis models and samples, apoptosis is not obvious in short-term stimulation of LPS *in vitro*, even though DNA damage had been observed in 48 h. The results were differed from some other cell types, for example, vascular endothelial cells,^21^ osteoblasts^33^ and airway epithelial cells,^34^ in which LPS induced oxidative stress and apoptosis. It suggested gingival fibroblast might possess the potential to resist toxic effects of LPS in the early stage of inflammation.

To verify the underlying mechanism, some DNA repair genes and apoptotic-associated proteins were examined. The mechanism resisting oxidative stress caused by LPS is likely related to the increased expression of Ogg1,^35^ Neil1^36^ and Rad50.^37,38^ These principal enzymes for DNA repair help to protect gingival fibroblast from early DNA damage.

Anti-apoptotic and pro-apoptotic proteins were detected. BCL-2, AKT, ERK and survivin are known as survival proteins. BCL-2 is considered to be an important anti-apoptotic protein, which protects DNA from fragmentation.^39^ Increase of BCL-2 may contribute to the anti-apoptotic effect of gingival fibroblast in this study. AKT is thought to be an anti-apoptotic protein. However, AKT could not protect against ROS-mediated cell death but rather sensitized cells to this cell death. In contrast to its ability to inhibit apoptosis induced by multiple apoptotic stimuli, decreased AKT could inhibit ROS-mediated apoptosis.^18^ In our study, decreased AKT may possess anti-apoptotic effects to resist cell death. ERK pathway has long been associated with cell growth, cell proliferation and survival.^40^ Nolan *et al.* reported that LPS signal transduction by ERK inhibits neutrophil apoptosis. Treatment of cells with ERK inhibitor would inhibit LPS signaled inhibition of neutrophil apoptosis.^41^ It indicated that ERK pathway promotes survival ability of gingival fibroblast. Survivin, a member of the inhibitors of apoptosis protein family, inhibits cell death through interference with both caspase-dependent and -independent cell apoptosis.^42^ Increase of survivin was an important factor to promote cell survival.

Conversely, p38, JNK and p53 are known as pro-apoptotic proteins. JNK and p38 MAPK are involved in the inflammatory response and mediated cell apoptosis.^43^ p53 is widely known as a tumor suppressor. One of the most dramatic responses to p53 activation is the induction of apoptosis.^44^ In periodontal diseases, p53 was observed in the inflammatory infiltrate.^29^ We have found that p38, JNK and p53 levels increased in LPS groups.

It is assumed that the anti-apoptotic effects and pro-apoptotic effects that were induced by LPS were balanced, and as a result, cell apoptosis was not induced by LPS in short time period. However, oxidative stress and DNA damage might induce other consequences, for example, cell senescence and autophagy. Cellular senescence in normal cells is mainly classified into two mutually exclusive types: one involves p16 and the other involves p21.^45^ In this study, p53–p21 pathway is expressed, but the level of p16 is not changed. LC3 is a specific marker reflecting autophagy and autophagy-related processes, including autophagic cell death.^46^ The results showed autophagy and p21-mediated senescence were involved. Autophagy shares similar upstream signaling pathways and stimulating factors with apoptosis. Sometimes it results in combined autophagy and apoptosis. Autophagy activation may inhibit cell apoptosis, but excessive autophagy may also cause autophagic cell death.^47^ The exact relationship among autophagy, senescence, apoptosis and LPS-induced oxidative stress needs further investigation.

The results help to explicate the consequence of LPS-induced oxidative stress in early gingivitis and chronic periodontitis. Gingival fibroblast possesses a short-term self-recovery time to resist toxic effect of LPS. During this time, the mRNA expressions of DNA repair enzymes were upregulated, the anti-apoptotic and pro-apoptotic effects were balanced, and cell damage tends to be reversible. The recovery time did not last long, as the DNA damage occurred after 48 h.

These results contribute to illustrate the mechanism that acute gingivitis could be curable when bacteria (dental plaque) are removed timely. Furthermore, the self-defense ability helps to keep gingiva ‘healthy’ during the interval of tooth brushing. Contrary to early and acute gingivitis, chronic and recurrent gingival inflammation induces cell damage, including DNA damage and cell apoptosis. It indicates periodontitis, which leads to irreversible damage to cells, even the periodontal tissue. The results confirm that early intervention to acute gingivitis is of most importance. If some measures, for example, increase the anti-apoptotic effect or DNA repair ability could be taken to prolong the self-recovery time, the periodontal tissue might be more resistant to inflammation.

In conclusion, gingival fibroblasts have potential to resist LPS-induced oxidative stress and cell apoptosis in periodontal diseases. The mechanism might be related to increased mRNA expression of DNA repair enzymes and balanced anti-apoptotic and pro-apoptotic proteins.

## Materials and Methods

### Cell culture

Mouse gingival fibroblast was cultured from gums obtained from 6- to 8-week-old female BalB/c mice. The gingival tissue was cut into pieces and cultured in Alpha modifications of Minimum Essential Medium (Sigma-Aldrich Co. LLC., St. Louis, MO, USA) with 10% fetal bovine serum plus 100 U/ml penicillin, and 100 *μ*g/ml streptomycin (all from Cambrex, Walkersville, MD, USA) at 37 °C with 5% CO_2_. Cells between passages 3–6 were used.

### Periodontitis mouse model

BalB/c and *Rag2^−/−^* mice (4- to 10-week-old female mice) were obtained from the Harlan Laboratories (Indianapolis, IN, USA). The mice were housed in a pathogen-free environment at the Cedars-Sinai Medical Center Animal Facility under the approval of the Institutional Animal Care and Use Committee (IACUC003638). The protocol ensured humane practices.

To establish the periodontitis mouse model, 10 *μ*g LPS (*Escherichia coli* LPS (O111 : B4; Sigma-Aldrich Co. LLC.)) in 10 *μ*l PBS or 10 *μ*l PBS vehicle were injected into mandibular buccal gingiva two times per week for 3 weeks.^14^ Mice were killed and gingival tissue was collected for histologic evaluation.

### Flow cytometry

Gingival fibroblasts were serum starved for 24 h then treated with 1 *μ*g/ml LPS for 24 h. For the apoptosis assay, cells were harvested and stained with Annexin V (eBioscience, San Diego, CA, USA) and 7-AAD (BD Biosciences, San Jose, CA, USA) according to the manufacturer’s instructions. Similarly, ROS activity was quantitated by DCFH-DA staining of serum-starved gingival fibroblasts treated with 1 *μ*g/ml LPS for 24 h. Cells were cultured with DCFH-DA for 20 min.

### Immunofluorescence staining

Immunofluorescent localization of *γ*H2AX in gingival fibroblasts was performed following 1 *μ*g/ml LPS treatment for 6, 24 and 48 h. Cells were fixed and incubated in PBS with 0.25% Triton X-100 (Sigma-Aldrich Co. LLC.) and 5% normal goat serum for 30 min, and subsequently incubated with anti-*γ*H2AX antibody (Trevigen Inc., Gaithersburg, MD, USA) overnight at 4 °C. Cells were washed and incubated with secondary antibody for 1 h. Cells were counterstained with DAPI and mounted. For DCFH-DA staining, serum-starved gingival fibroblasts were treated with 1 *μ*g/ml LPS for 24 h. Cells were cultured with DCFH-DA for 20 min. The fluorescent images were taken using a fluorescence microscope (Olympus Fsx100, Olympus Corporation, Tokyo, Japan).

### Western blot

Gingival fibroblasts were serum starved for 24 h and then treated with 1 *μ*g/ml LPS for 24 h. Proteins were detected following western blot procedure. The following proteins were detected using antibodies (Cell Signaling Technology, Inc., Danvers, MA, USA): anti-caspase-3, anti-BCL2, anti-AKT (phosphor Ser473), anti-ERK1/2 (phosphorylated Thr202/Tyr204), anti-SAPK/JNK (phosphorylated Thr183/Tyr185), anti-p38 (phosphorylated Thr180/Tyr182) and anti-survivin. Anti-p53, anti-p21, anti-p16 and anti-*β*-actin antibodies from Santa Cruz Biotechnology, Inc. (Santa Cruz, CA, USA) were used. Anti-LC3 from Novus Biologicals, LLC (Littleton, CO, USA) were used.

### RT-PCR

Gingival fibroblasts were serum starved for 24 h then treated with 1 *μ*g/ml LPS for 24 h.

Total mRNA was isolated using RNase mini kit (Qiagen, Hilden, Germany). Approximately, 1 *μ*g of total RNA was used to synthesize cDNA with (Bio-Rad Laboratories, Inc., Hercules, CA, USA). mRNA expression was determined by RT-PCR, which was performed on Bio-Rad's S1000 Thermal Cycler (Bio-Rad Laboratories) using GoTaq Green Master Mix (VWR International, Radnor, PA, USA). Primer sequences used are shown in Supplementary Table 1.

### Clinical samples, immunohistochemistry and analysis

Human gingival tissues obtained from crown lengthening surgery, eruption aiding surgery (normal gingival tissues) and gingivectomy (periodontitis tissues) were collected at West China Hospital of Stomatology, Sichuan University (Chengdu, Sichuan, China). The protocol was reviewed and subjects’ rights have been protected by the Institutional Ethics Committee of West China Hospital of Stomatology (WCHSIRB-ST-2014-091). All subjects provided informed written consent. Immunohistochemical detection was conducted using antibodies 8-OHdG (Abcam, Cambridge, MA, USA) and TUNEL (Thermo Fisher Scientific Inc., Waltham, MA, USA). Images were captured with Aperio AT2 (Leica Biosystems, Wetzlar, Germany). The number of immunoreactive cells was calculated and evaluated as the percentage (0–100%) of positive cells.

### Statistical analysis

Data were expressed as mean±S.E.M. The statistical significance of differences among groups was assessed using Student’s *t*-test and one-way ANOVA with LSD post-hoc test by SPSS 16.0 (IBM Corp., New York, NY, USA). A difference was considered significant when *P*<0.05.

## Abbreviations

LPS: lipopolysaccharide
ROS: reactive oxygen species
GF: gingival fibroblast
8-OHdG: 8-hydroxy-2′-deoxyguanosine
*γ*H2AX: gamma-H2AX
TUNEL: TdT-mediated dUTP nick end labeling
DCFH-DA: 2′,7′-Dichlorofluorescein diacetate.

## References

[bib1] Page RC , Offenbacher S , Schroeder HE , Seymour GJ , Kornman KS . Advances in the pathogenesis of periodontitis: summary of developments, clinical implications and future directions. Periodontol 2000 1997; 14: 216–248. 9567973 10.1111/j.1600-0757.1997.tb00199.x

[bib2] Gorąca A , Huk-Kolega H , Kleniewska P , Piechota-Polańczyk A , Skibska B . Effects of lipoic acid on spleen oxidative stress after LPS administration. Pharmacol Rep 2013; 65: 179–186. 23563036 10.1016/s1734-1140(13)70976-9

[bib3] Melo ES , Barbeiro HV , Ariga S , Goloubkova T , Curi R , Valasco IT et al. Immune cells and oxidative stress in the endotoxin tolerance mouse model. Braz J Med Biol Res 2010; 43: 57–67. 20027485 10.1590/s0100-879x2009007500027

[bib4] Sanikidze TV , Tkhilava NG , Papava MB , Datunashvili IV , Gongadze MT , Gamrekelashvili DD et al. Role of free nitrogen and oxygen radical in the pathogenesis of lipopolysaccharide-induced endotoxemia. Bull Exp Biol Med 2006; 141: 211–215. 16984099 10.1007/s10517-006-0130-3

[bib5] Bykov I , Ylipaasto P , Erola L , Lindros KO . Phagocytosis and LPS stimulated production of cytokines and prostaglandin E2 is different in Kupffer cells isolated from the periportal or perivenous liver region. Scand J Gastroenterol 2003; 38: 1256–1261. 14750646 10.1080/00365520310007116

[bib6] Knight JA . Diseases related to oxygen-derived free radicals. Ann Clin Lab Sci 1995; 25: 111–121. 7785961

[bib7] Kratzer E , Tian Y , Sarich N , Wu T , Meliton A , Leff A et al. Oxidative stress contributes to lung injury and barrier dysfunction via microtubule destabilization. Am J Respir Cell Mol Biol 2012; 47: 688–697. 22842495 10.1165/rcmb.2012-0161OC PMC3547103

[bib8] Tomofuji T , Azuma T , Kusano H , SanbeT , Ekuni D , Tamaki N et al. Oxidative damage of periodontal tissue in the rat periodontitis model: effects of a high-cholesterol diet. FEBS Lett 2006; 580: 3601–3604. 16750199 10.1016/j.febslet.2006.05.041

[bib9] Maruyama T , Tomofuji T , Endo Y , Irie K , Azuma T , Ekuni D et al. Supplementation of green tea catechins in dentifrices suppresses gingival oxidative stress and periodontal inflammation. Arch Oral Biol 2011; 56: 48–53. 20869695 10.1016/j.archoralbio.2010.08.015

[bib10] Cleaver JE . γH2Ax: biomarker of damage or functional participant in DNA repair “all that glitters is not gold!”. Photochem Photobiol 2011; 87: 1230–1239. 21883247 10.1111/j.1751-1097.2011.00995.x

[bib11] Banerjee J , Mishra R , Li X , Jackson RS 2nd , Sharma A , Bhowmick NA . A reciprocal role of prostate cancer on stromal DNA damage. Oncogene 2014; 33: 4924–4931. 24141771 10.1038/onc.2013.431 PMC4121379

[bib12] Bishayee K , Paul A , Ghosh S , Sikdar S , Mukherjee A , Biswas R et al. Condurango-glycoside-A fraction of Gonolobus condurango induces DNA damage associated senescence and apoptosis via ROS-dependent p53 signalling pathway in HeLa cells. Mol Cell Biochem 2013; 382: 173–183. 23807740 10.1007/s11010-013-1732-5

[bib13] Dimri GP . What has senescence got to do with cancer? Cancer Cell 2005; 7: 505–512. 15950900 10.1016/j.ccr.2005.05.025 PMC1769521

[bib14] Yokoyama M , Ukai T , Ayon Haro ER , Kishimoto T , Yoshinaga Y , Hara Y . Membrane-bound CD40 ligand on T cells from mice injected with lipopolysaccharide accelerates lipopolysaccharide-induced osteoclastogenesis. J Periodontal Res 2011; 46: 464–474. 21521224 10.1111/j.1600-0765.2011.01362.x

[bib15] Valavanidis A , Vlachogianni T , Fiotakis C . 8-Hydroxy-2'-deoxyguanosine (8-OHdG): a critical biomarker of oxidative stress and carcinogenesis. J Environ Sci Health C Environ Carcinog Ecotoxicol Rev 2009; 27: 120–139. 19412858 10.1080/10590500902885684

[bib16] Haldar S , Dru C , Choudhury D , Mishra R , Fernandez A , Biondi S et al. Inflammation and pyroptosis mediate muscle expansion in an interleukin-1β (IL-1β)-dependent manner. J Biol Chem 2015; 290: 6574–6583. 25596528 10.1074/jbc.M114.617886 PMC4358290

[bib17] Shinkai Y , Rathbun G , Lam KP , Oltz EM , Stewart V , Mendelsohn M et al. RAG-2-deficient mice lack mature lymphocytes owing to inability to initiate V(D)J rearrangement. Cell 1992; 68: 855–867. 1547487 10.1016/0092-8674(92)90029-c

[bib18] Nogueira V , Park Y , Chen CC , Xu PZ , Chen ML , Tonic I et al. Akt determines replicative senescence and oxidative or oncogenic premature senescence and sensitizes cells to oxidative apoptosis. Cancer Cell 2008; 14: 458–470. 19061837 10.1016/j.ccr.2008.11.003 PMC3038665

[bib19] Meng Z , Yan C , Deng Q , Gao DF , Niu XL . Curcumin inhibits LPS-induced inflammation in rat vascular smooth muscle cells in vitro via ROS-relative TLR4-MAPK/NF-κB pathways. Acta Pharmacol Sin 2013; 34: 901–911. 23645013 10.1038/aps.2013.24 PMC4002611

[bib20] Yang YI , Jung SH , Lee KT , Choi JH . 8,8'-Bieckol, isolated from edible brown algae, exerts its anti-inflammatory effects through inhibition of NF-κB signaling and ROS production in LPS-stimulated macrophages. Int Immunopharmacol 2014; 23: 460–468. 25261704 10.1016/j.intimp.2014.09.019

[bib21] Li J , He J , Yu C . Chitosan oligosaccharide inhibits LPS-induced apoptosis of vascular endothelial cells through the BKCa channel and the p38 signaling pathway. Int J Mol Med 2012; 30: 157–164. 22469656 10.3892/ijmm.2012.954

[bib22] Kim JC , Lee YH , Yu MK , Lee NH , Park JD , Bhattarai G et al. Anti-inflammatory mechanism of PPARγ on LPS-induced pulp cells: role of the ROS removal activity. Arch Oral Biol 2012; 57: 392–400. 21996491 10.1016/j.archoralbio.2011.09.009

[bib23] Gölz L , Memmert S , Rath-Deschner B , Jäger A , Appel T , Baumgarten G et al. LPS from P. gingivalis and hypoxia increases oxidative stress in periodontal ligament fibroblasts and contributes to periodontitis. Mediators Inflamm 2014; 2014: 986264. 25374447 10.1155/2014/986264 PMC4211166

[bib24] Okamura DM , Himmelfarb J . Tipping the redox balance of oxidative stress in fibrogenic pathways in chronic kidney disease. Pediatr Nephrol 2009; 24: 2309–2319. 19421784 10.1007/s00467-009-1199-5

[bib25] Ekuni D , Tomofuji T , Tamaki N , Sanbe T , Azuma T , Yamanaka R et al. Mechanical stimulation of gingiva reduces plasma 8-OHdG level in ratperiodontitis. Arch Oral Biol 2008; 53: 324–329. 18031711 10.1016/j.archoralbio.2007.10.005

[bib26] Ekuni D , Endo Y , Irie K , Azuma T , Tamaki N , Tomofuji T et al. Imbalance of oxidative/anti-oxidative status induced by periodontitis is involved in apoptosis of rat submandibular glands. Arch Oral Biol 2010; 55: 170–176. 20035925 10.1016/j.archoralbio.2009.11.013

[bib27] Sinha K , Das J , Pal PB , Sil PC . Oxidative stress: the mitochondria-dependent and mitochondria-independent pathways of apoptosis. Arch Toxicol 2013; 87: 1157–1180. 23543009 10.1007/s00204-013-1034-4

[bib28] Kannan K , Jain SK . Oxidative stress and apoptosis. Pathophysiology 2000; 7: 153–163. 10996508 10.1016/s0928-4680(00)00053-5

[bib29] Manucha W , Vallés PG . Apoptosis modulated by oxidative stress and inflammation during obstructive nephropathy. Inflamm Allergy Drug Targets 2012; 11: 303–312. 22533548 10.2174/187152812800958997

[bib30] Gamonal J , Bascones A , Acevedo A , Blanco E , Silva A . Apoptosis in chronic adult periodontitis analyzed by in situ DNA breaks, electron microscopy, and immunohistochemistry. J Periodontol 2001; 72: 517–525. 11338305 10.1902/jop.2001.72.4.517

[bib31] Lucas H , Bartold PM , Dharmapatni AA , Holding CA , Haynes DR . Inhibition of apoptosis in periodontitis. J Dent Res 2010; 89: 29–33. 19948942 10.1177/0022034509350708

[bib32] Fu YW , He HB . Apoptosis of periodontium cells in streptozototocin- and ligature-induced experimental diabetic periodontitis in rats. Acta Odontol Scand 2013; 71: 1206–1215. 23294164 10.3109/00016357.2012.757638

[bib33] Ansari N , Khodagholi F , Ramin M , Amini M , Irannejad H , Dargahi L et al. Inhibition of LPS-induced apoptosis in differentiated-PC12 cells by new triazine derivatives through NF-κB-mediated suppression of COX-2. Neurochem Int 2010; 57: 958–968. 20946929 10.1016/j.neuint.2010.10.002

[bib34] Cho IH , Gong JH , Kang MK , Lee EJ , Park JH , Park SJ et al. Astragalin inhibits airway eotaxin-1 induction and epithelial apoptosis through modulatingoxidative stress-responsive MAPK signaling. BMC Pulm Med 2014; 29: 122. 10.1186/1471-2466-14-122 PMC4118077 25069610

[bib35] Ba X , Aguilera-Aguirre L , Rashid QT , Bacsi A , Radak Z , Sur S et al. The role of 8-oxoguanine DNA glycosylase-1 in inflammation. Int J Mol Sci 2014; 15: 16975–16997. 25250913 10.3390/ijms150916975 PMC4200771

[bib36] Hegde ML , Hegde PM , Arijit D , Boldogh I , Mitra S . Human DNA glycosylase NEIL1's interactions with downstream repair proteins is critical for efficient repair of oxidized DNA base damage and enhanced cell survival. Biomolecules 2012; 2: 564–278. 23926464 10.3390/biom2040564 PMC3733129

[bib37] Ghodke I , Muniyappa K . Processing of DNA double-stranded breaks and intermediates of recombination and repair by Saccharomyces cerevisiae Mre11 and its stimulation by Rad50, Xrs2, and Sae2 proteins. J Biol Chem 2013; 288: 11273–11286. 23443654 10.1074/jbc.M112.439315 PMC3630844

[bib38] He J , Shi LZ , Truong LN , Lu CS , Razavian N , Li Y et al. Rad50 zinc hook is important for the Mre11 complex to bind chromosomal DNA double-stranded breaks and initiate various DNA damage responses. J Biol Chem 2012; 287: 31747–31756. 22833675 10.1074/jbc.M112.384750 PMC3442509

[bib39] Hardwick JM , Soane L . Multiple functions of BCL-2 family proteins. Cold Spring Harb Perspect Biol 2013; 5: a008722. 23378584 10.1101/cshperspect.a008722 PMC3552500

[bib40] Ballif BA , Blenis J . Molecular mechanisms mediating mammalian mitogen-activated protein kinase (MAPK) kinase (MEK)-MAPK cell survival signals. Cell Growth Differ 2001; 12: 397–408. 11504705

[bib41] Nolan B , Duffy A , Paquin L , De M , Collette H , Graziano CM et al. Mitogen-activated protein kinases signal inhibition of apoptosis in lipopolysaccharide-stimulated neutrophils. Surgery 1999; 126: 406–412. 10455914

[bib42] Cheung CH , Cheng L , Chang KY , Chen HH , Chang JY . Investigations of survivin: the past, present and future. Front Biosci (Landmark Ed) 2011; 16: 952–961. 21196211 10.2741/3728

[bib43] Ki YW , Park JH , Lee JE , Shin IC , Koh HC . JNK and p38 MAPK regulate oxidative stress and the inflammatory response in chlorpyrifos-induced apoptosis. Toxicol Lett 2010; 218: 235–245. 10.1016/j.toxlet.2013.02.003 23416140

[bib44] Amaral JD , Xavier JM , Steer CJ , Rodrigues CM . The role of p53 in apoptosis. Discov Med 2010; 9: 145–152. 20193641

[bib45] Beausejour CM , Krtolica A , Galimi F , Narita M , Lowe SW , Yaswen P et al. Reversal of human cellular senescence: roles of the p53 and p16 pathways. EMBO J 2003; 22: 4212–4222. 12912919 10.1093/emboj/cdg417 PMC175806

[bib46] Tanida I , Ueno T , Kominami E . LC3 and autophagy. Methods Mol Biol 2008; 445: 77–88. 18425443 10.1007/978-1-59745-157-4_4

[bib47] Maiuri MC , Zalckvar E , Kimchi A , Kroemer G . Self-eating and self-killing: crosstalk between autophagy and apoptosis. Nat Rev Mol Cell Biol 2007; 8: 741–752. 17717517 10.1038/nrm2239

