# Supplementary figures and images for: Gingival fibroblasts resist apoptosis in response to oxidative stress in a model of periodontal diseases
Source: Cell Death Discov. 2015 Nov 9;1:15046–. doi: 10.1038/cddiscovery.2015.46 (PMC4979524; doi:10.1038/cddiscovery.2015.46)

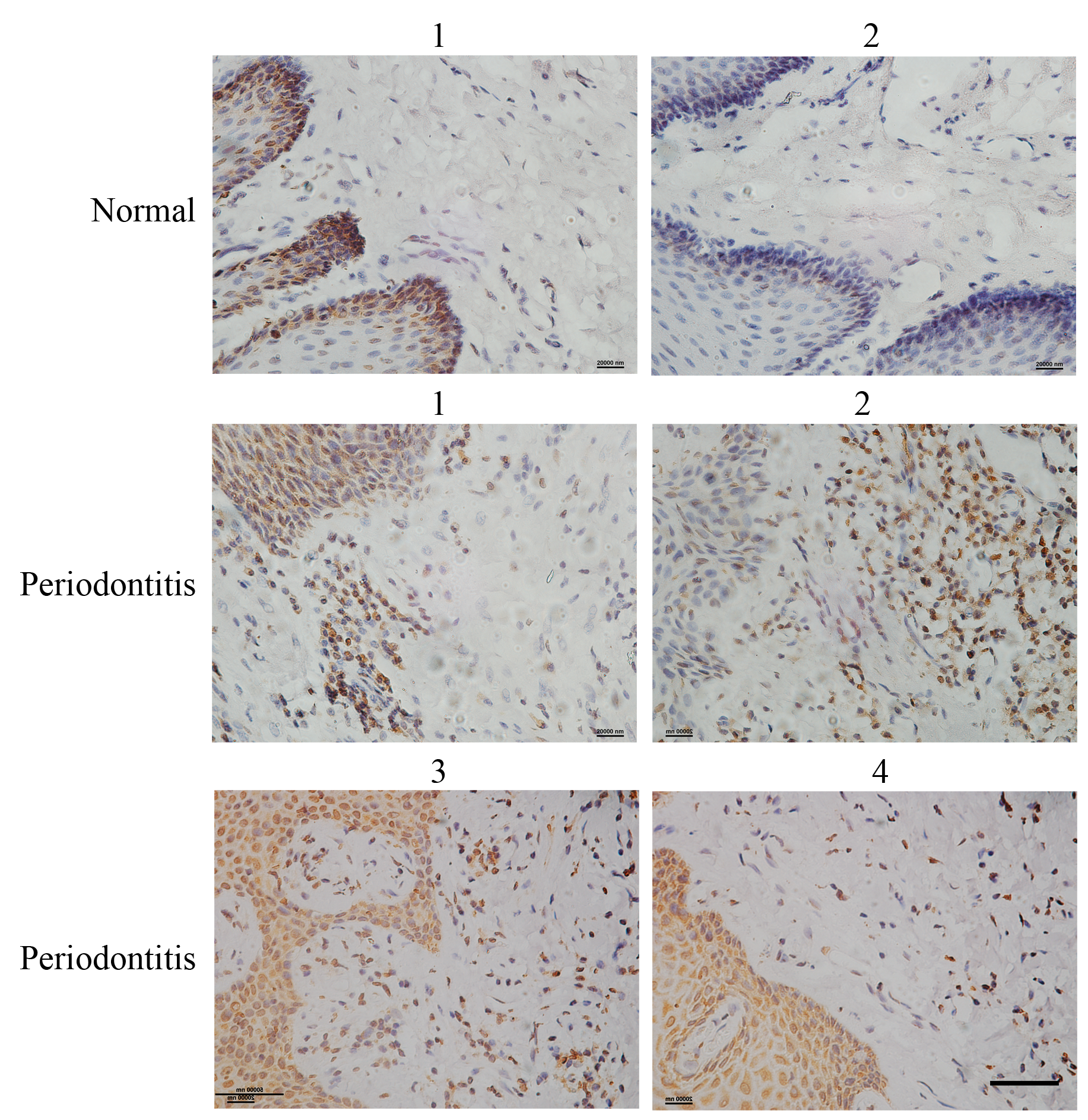

Supplement: Supplementary Figure 1 [file cddiscovery201546-s1.tiff]
