# Supplementary material for: Gingival fibroblasts resist apoptosis in response to oxidative stress in a model of periodontal diseases
Source: Cell Death Discov. 2015 Nov 9;1:15046–. doi: 10.1038/cddiscovery.2015.46 (PMC4979524; doi:10.1038/cddiscovery.2015.46)
Supplement: Supplementary Information [file cddiscovery201546-s2.doc]

**Supplementary figure legends**

**Supplementary Figure 1.**Paraffin-embeddedhuman gingival samples were stained for 8-OHdG by IHC. 2 healthy and 4 periodontitis specimens were shown. Bar, 50 μm

**Supplementary Table 1.Primers for RT-PCR**

| Primer | Forward | Reverse |
| --- | --- | --- |
| Ogg1 | 5’-GCG AGA GGC TTT CCT ACG AG-3’ | 5’-AGT CCT AAA GCC TCG CAC AC-3’ |
| Neil1 | 5’-TGG TAT TTG GTG GGT GTG TGG AGA-3’ | 5’-ATC CTG ACA TCC CAA AGC GGA AGA-3’ |
| Rad50 | 5’-TGGTGA CCA TGACAG AAC TGG ACA-3’ | 5’-TTG CGA CTG GGT ATG TGA GGT GAA-3’ |
| β-actin | 5’-GTG GGC CGC TCT AGG CAC CA-3’ | 5’-CGG TTG GCC TTA GGG TTC AGG-3’ |
